# CIP2A regulates cell proliferation via the AKT signaling pathway in human lung cancer

**DOI:** 10.3892/or.2014.3375

**Published:** 2014-08-01

**Authors:** NINGJING LEI, BO PENG, JIAN-YING ZHANG

**Affiliations:** Border Biomedical Research Center and Department of Biological Sciences, The University of Texas at El Paso, El Paso, TX 79968, USA

**Keywords:** CIP2A, cell proliferation, lung cancer, AKT signaling pathway

## Abstract

Cancerous inhibitor of PP2A (CIP2A) is an intracellular endogenous protein phosphatase 2A (PP2A) inhibitor with oncogenic activities. Initially identified as a tumor-associated antigen (TAA) in gastric and liver cancer patients, CIP2A was overexpressed in a variety of cancer types. The overexpression of CIP2A in cancer cells is associated with increased cell proliferation. However, the mechanism of CIP2A in cancer cell proliferation remains poorly understood. In the present study, we reported that CIP2A can regulate AKT phosphorylation at S473 under growth factor stimulation and our results also showed that CIP2A may promote cell proliferation through the AKT signaling pathway. Notably, depletion of CIP2A did not induce a global change of AKT phosphatase activity, which indicated that CIP2A may recognize specific AKT targets and play certain roles in the signaling pathway. In addition, we detected that CIP2A expression was associated with mTOR phosphorylation. Our further analysis corroborated the relationship between CIP2A and AKT-mTOR signaling pathway. Therefore, our study addressed a novel role of CIP2A in mediating cancer progression through interacting with the AKT-mTOR signaling pathway.

## Introduction

Lung cancer is characterized by uncontrolled cell growth in the lungs, and is by far the leading cause of cancer-related mortality in both men and women in the USA (1). Based on the relative size of the cancer cells as examined under a microscope, lung cancer can be classified as non-small cell lung cancer (NSCLC) or small cell lung cancer (SCLC) (2). The etiological factors for lung cancer are complex and it is believed that lung cancer, similar to other types of cancer, is initiated by activation of oncogenes and inactivation of tumor suppressor genes (3). Current understanding of the molecular mechanism of lung cancer showed that the genetic mutations contribute to the oncogenesis and development of cancer. Mutated oncogenes, such as epidermal growth factor receptor (EGFR), c-myc, K-ras, anaplastic lymphoma kinase (ALK) and phosphatidylinositol 3-kinases (PI3K), have been found to contribute to the formation of NSCLC (4–7).

Cancerous inhibitor of PP2A (CIP2A) is a newly characterized oncogenic protein. CIP2A was initially identified as a tumor-associated autoantigen in gastric and liver cancer (8). The high frequency of autoantibodies to CIP2A detected in the sera of cancer patients makes it a promising candidate for biomarker development. The expression of CIP2A was only present in embryonic development but was silenced from all tissues soon after birth. The function of CIP2A was not discovered until 2007 during the search of protein phosphatase 2A (PP2A) interacting proteins in human cervical cancer cell line HeLa (9). PP2A complexes function by inhibiting activity of several important oncogenic signaling pathways as tumor suppressors (10). The PP2A holoenzyme consists of the scaffold A subunit, various regulatory B subunits and the catalytic C subunit (11–13). Junttila *et al* found that CIP2A was co-purified with the A subunit of PP2A (9). The interaction between CIP2A and PP2A led to the discovery of the inhibitory effect of CIP2A to the phosphatase activity of PP2A. Therefore, the finding that CIP2A is an endogenous PP2A inhibitor may greatly expand our understanding of cellular transformation in cancer progression.

Previous studies have shown that the overexpression of CIP2A is detected in various types of tumors, from solid tumors to hematological malignancies, including breast, gastric, head and neck, lung cancer and leukemia, and subsequently it was known as an oncofetal protein (14–17). Additional functions of CIP2A in cancer progression are still under investigation. A recent report showed that CIP2A could protect the hepatocellular carcinoma (HCC) cell lines from bortezomib-induced apoptosis by inhibiting phosphor-AKT-associated PP2A phosphatase activity (18). The knockdown of CIP2A via siRNA thus sensitizes HCC cell lines to bortezomib treatment. The present study suggests that CIP2A is able to regulate the phosphorylation of protein kinase B (PKB)/AKT in response to the treatment of chemotherapeutic drugs and this protein may have more functions unknown in tumorigenesis. However, whether the interplay between CIP2A and phospho-AKT plays a role in cancer progression, and how CIP2A facilitates AKT-mediated resistance to chemotherapy, remains unresolved.

In the present study, we sought to elucidate the association between CIP2A and the AKT signaling pathway in regulating cell proliferation. The findings obtained from this study will expand the understanding of protein-substrate interaction and therefore direct the cancer drug design. At the same time, we focused on the mechanism of AKT phosphorylation to further investigate how CIP2A is involved in the PI3K/AKT/mTOR pathway, which is critical to cancer progression.

## Materials and methods

### Cell culture and transfections

The three lung cancer cell lines, NCI-H838, NCI-H1299 and NCI-H460 were purchased from American Type Culture Collection (ATCC, Manassas, VA, USA). All cell lines were cultured in RPMI-1640 medium containing 10% fetal bovine serum (FBS) (both from Life Technologies, Carlsbad, CA, USA) at 37°C with 5% CO_2_.

Transient transfection of pcDNA3.1 control or pcDNA3.1 containing CIP2A cDNA (a gift from Dr Westermarck, Finland) into the H1299 lung cancer cell line was performed with Lipofectamine LTX (Life Technologies) according to the manufacturer’s protocol.

### Production of lentivirus encoding CIP2A short hairpin RNA

Five CIP2A short hairpin RNAs, ligated in pLKO.1 vector, were obtained from Open Biosystems (Huntsville, AL, USA). The knockdown efficiency of these shRNAs was evaluated in H1299 cells by western blotting and two of them showing the higher knockdown efficiency were chosen to produce lentivirus. Lentivirus was produced by co-transfection of pLKO.1 control (ligated with scramble sequence) or other pLKO.1-derived vector with pMD2.G and pCMV-VSVG (both from Addgene, Cambridge, MA, USA) into HEK293T packaging cell lines. The supernatants containing lentivirus of HEK293T were harvested at 36 and 72 h post-transfection. Supernatants were pooled, centrifuged to remove cells and then filtered through a 0.45 μm low protein binding filter. Cells were plated in monolayer at different densities and infected with lentivirus constructs using 8 ng/ml polybrene (Sigma, St. Louis, MO, USA). The stable cell lines were selected in the presence of 1 μg/ml puromycin (Sigma) for two weeks.

### Cell proliferation assay

To compare cell proliferation in cells with CIP2A knockdown, the MTT assay was performed. The shRNA for CIP2A lentivirus-transduced and control shRNA-transduced cells was plated quadruplicate in 96-well plates at a density of 3×10^3^/100 μl medium for each well. After a 3-day culture, 15 μl of the dye solution was added to each well and incubated at 37°C for another 4 h. At the end of the incubation, 100 μl stop solution was added to each well and colorimetric absorbance was read in the SpectraMax Plus (Molecular Devices) at 570 nm. Cell free medium was used as mock control.

### PP2A phosphatase activity assay

Measurement of PP2A phosphatase activity was performed by using the RediPlate™ 96 EnzChek^®^ Serine/Threonine Phosphatase Assay kit (Life Technologies). Cell lysates were prepared by using low detergent buffer (1% Nonidet P-40, 10 mM HEPES, 150 mM NaCl, 10% glycerol, 1 mM PMSF, and complete protease inhibitor cocktail). A total of 50 μl cell lysates were incubated with 1X PP2A phosphatase reaction buffer for 30 min at 37°C. Fluorescence intensity was measured using excitation at 355 nm and emission at 485 nm. The fluorescence intensity was normalized to the expression level of PP2A catalytic domain.

### Western blot analysis

Cells were plated in 6-well tissue culture plates at 80% confluence and incubated overnight. Cell lysates were obtained from transduced cells using cold radioimmunoprecipitation assay buffer [20 mmol/l Tris-HCl (pH 8.0), 100 mmol/l NaCl, 10% glycerol, 1% NP40, 0.5% sodium deoxycholate]. Twenty micrograms of protein mixture were separated on 10% SDS-PAGE gels and wet transferred to nitrocellulose membrane (GE Healthcare Life Sciences) and then blocked for 1 h at room temperature in TBS-T buffer [50 mmol/l Tris-HCl (pH 7.5), 150 mmol/l NaCl, 0.1% Tween-20] containing 5% non-fat milk. Membranes were then incubated overnight at 4°C or 1 h at room temperature with the respective primary antibodies: anti-CIP2A (1:500), and anti-actin (1:1,000; Santa Cruz Biotechnology, Santa Cruz, CA, USA), phosphor-mTOR (1:1,000), mTOR (1:1,000), phospho-AKT (S473) (1:1,000), (Cell Signaling Technology, Danvers, MA, USA). Anti-mouse or anti-rabbit secondary antibody-conjugated to horseradish peroxidase was used to visualize the stained bands with an enhanced chemiluminescence visualization kit (both from Santa Cruz Biotechnology).

### Statistical analysis

All values from *in vitro* assays are expressed as means ± SD or SEM of at least three independent experiments or replicates. p-values were calculated with the two-tailed Student’s t-test. A p-value <0.05 was considered to indicate a statistically significant difference.

## Results

### CIP2A regulates lung cancer cell proliferation

**Figure 1:**
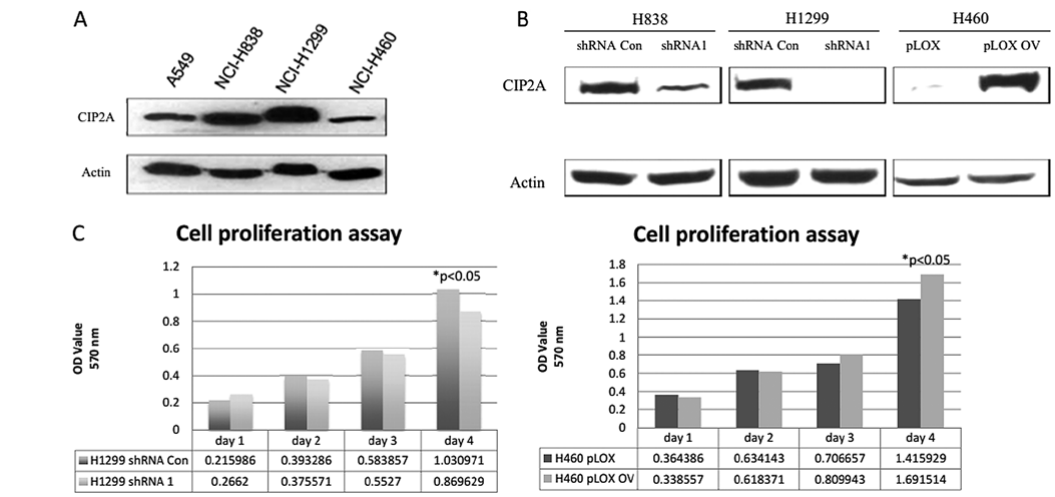
CIP2A expression and cell proliferation assay. (A) CIP2A expression in NSCLC was examined in four cell lines, A549, H838, H1299 and H460, by using mouse anti-CIP2A monoclonal antibody (1:2,000). (B) Cells transduced with shRNA control or shRNA CIP2A or pLOX or pLOX-CIP2A were evaluated in three cell lines (H838, H1299 and H460). (C) Cell proliferation assay was performed in H1299 cell line transduced with shRNA control or shRNA CIP2A and H460 cell line transduced with pLOX or pLOX-CIP2A. Five thousand cells of each group were plated sextuplet in 96-well plates and grew for the indicated times (day 1, 2, 3 and 4). Cell proliferation assay was performed by incubation of cell culture with MTT for 4 h and the absorbance was measured at 570 nm in a colorimetric meter.

To establish the role of CIP2A in the cell proliferation of lung cancer cells, we first examined the expression of CIP2A in four different lung cancer cell lines, A549, NCI-H838 (H838), NCI-H1299 (H1299) and NCI-H460 (H460). The four NSCLC cell lines expressed apparently different levels of endogenous CIP2A (Fig. 1A). We generated five cell lines with wild-type CIP2A (pcDNA3.1 and shRNA control), deficient CIP2A via stable transfection of two independent shRNAs (CIP2A shRNA1 and CIP2A shRNA2) or overexpressed CIP2A (pcDNA3.1+CIP2A) via transient transfection. The CIP2A expression level in knockdown or overexpression cell lines was confirmed by western blotting (Fig. 1B). The knockdown of CIP2A caused the decreased cell proliferation while over-expression of CIP2A led to the increased cell proliferation rate (Fig. 1C). These data suggested the function of CIP2A in promoting lung cancer cell proliferation.

### CIP2A regulates cell proliferation through epidermal growth factor (EGF)-stimulated AKT signaling pathway

**Figure 2:**
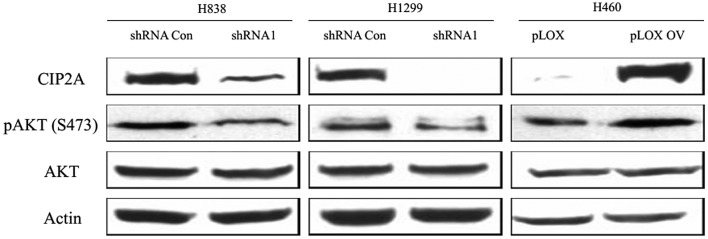
CIP2A regulates AKT phosphorylation in lung cancer cells. Phosphorylation of AKT between cells transduced with shRNA control or shRNA CIP2A or pLOX or pLOX-CIP2A was evaluated in three cell lines (H838, H1299 and H460). Equal number of cells was starved for 24 h and then stimulated with EGF for 30 min. Cells were harvested, lysed in 1X Laemmli sample buffer, resolved on 10% SDS-PAGE, transferred to nitrocellouse membrane and probed with rabbit anti-phospho-AKT (1:1,000). CIP2A to actin ratio was analyzed with densitometry.

One of the most important growth factors associated with lung cancer is the EGF (19). Upon EGF binding to its receptor, the downstream MAPKs are activated leading ultimately to cell proliferation. CIP2A can decrease AKT associated PP2A phosphatase activity. In response to EGF stimulation, AKT became phosphorylated at S473, a residue indicated in enzymatic activity and substrate specificity. We either depleted CIP2A using shRNA or elevated CIP2A expression level via ectopic overexpression in these three cell lines. As shown in Fig. 2, the knockdown of CIP2A in H1299 and H838 caused the hypophosphorylation of AKT while overexpression of CIP2A in H460 caused the hyperphosphorylation of AKT. The total amount of AKT was unaffected by the level of CIP2A. Therefore, CIP2A regulates AKT phosphorylation in lung cancer cells.

**Figure 3:**
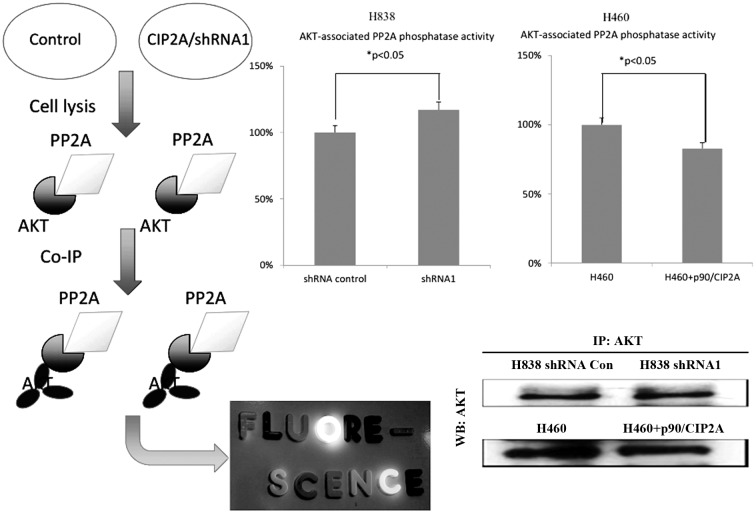
CIP2A modulates AKT-associated PP2A phosphatase activity. AKT-associated PP2A phosphatase activity was evaluated in two lung cancer cell lines, H838 and H460, with either CIP2A depletion (left panel) or overexpression (right panel). Western blot results showed the amount of AKT in the 5% of the immunoprecipitates in both of H1299 and H460, which is used to normalize the PP2A phosphatase activity.

To investigate whether the downregulated AKT phosphorylation is due to the elevated PP2A phosphatase activity, we analyzed the AKT-associated PP2A phosphatase activity by performing the PP2A phosphatase activity assay (Fig. 3). The knockdown of CIP2A increased the AKT-associated PP2A phosphatase activity, while overexpression of CIP2A downregulated AKT-associated PP2A phosphatase activity. The results indicated that the regulation of CIP2A on AKT-phosphorylation is associated with PP2A phosphatase activity.

**Figure 4:**
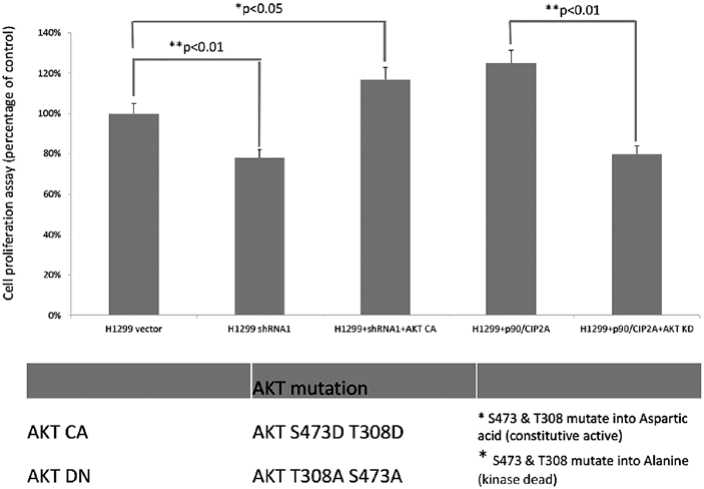
CIP2A promotes cell proliferation through AKT signaling. Cell proliferation was measured in cell lines transfected or co-transfected with either shRNA control, CIP2A shRNA (shRNA1), shRNA1 and AKT CA, pCDNA3.1+CIP2A or pcDNA3.1-CIP2A and AKT KD. Five thousand transfected cells were plated sextuplet in 96-well plates and cell proliferation was measured 48 h post-transfection. After 4 h incubation with MTT, cell proliferation was stopped with Stop solution and absorption was measured at 570 nm in a colorimetric meter.

Since AKT is an important regulator in cancer cell proliferation and tumor cell growth, we tested whether CIP2A-mediated cell proliferation can be partly attributed to the AKT phosphorylation. In the H1299 cell line, the knockdown of CIP2A decreased cell proliferation to 78%. However, the introduction of constitutively active AKT (AKT CA) rescued the decreased cell proliferation to 119%. Furthermore, the expression of dominant-negative AKT (AKT KD) therefore decreased the cell proliferation imposed by the CIP2A overexpression (123 vs. 81%). The introduction of these two AKT mutations provided direct evidence that CIP2A promoted cell proliferation through the regulation of AKT phosphorylation (Fig. 4).

### CIP2A regulates mTOR phosphorylation and its downstream effectors

Mammalian target of rapamycin (mTOR) is a downstream target of AKT signaling pathway, which transmits the signaling to promote protein synthesis and cell growth. mTOR functions by two different complexes, mTOR complex 1 (mTORC1) and complex 2 (mTORC2). mTORC1 plays various roles in regulating cell growth, proliferation and survival. It phosphorylates two downstream factors S6 kinase and eukaryotic initiation factor 4E-binding protein 1 (4E-BP1). Functions of mTORC2 have not been well studied, but it is suggested to be part of downstream branch in the PI3K/AKT pathway (20).

**Figure 5:**
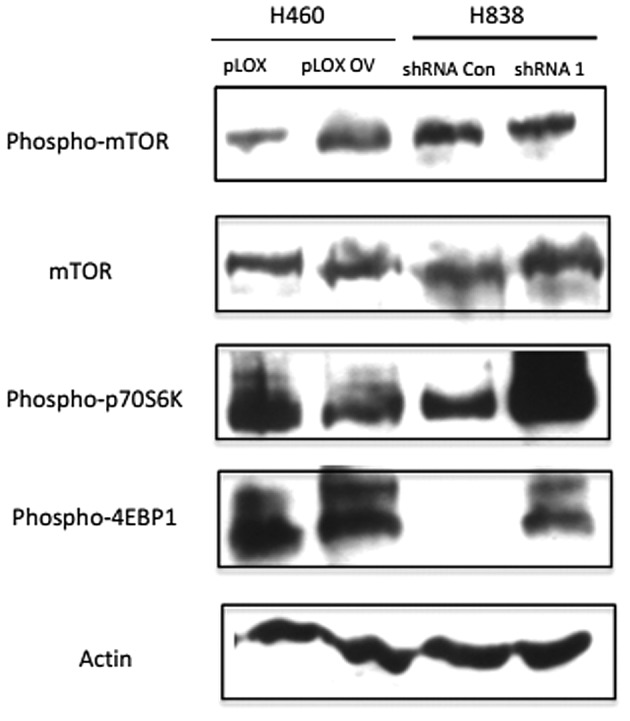
CIP2A modulates mTOR phosphorylation and the expression level of mTOR downstream substrates. Phosphorylation of mTOR phosphorylation and its downstream effectors, eukaryotic initiation factor 4E-binding protein 1 (4E-BP1) and P70 ribosomal S6 kinase 1 (P70S6K1) were analyzed in H838 and H460 cancer cell lines with either CIP2A depletion or CIP2A overexpression. Cells were starved, stimulated with EGF (100 ng/ml) for 30 min and lysed with 1X Laemmli buffer. Proteins were resolved on 10% SDS-PAGE gel. Phosphorylation of mTOR was analyzed with rabbit anti-phospho-mTOR (S2441) (1:1,000) and normalized to the expression level of actin.

To test whether CIP2A-regulated AKT phosphorylation would affect this signaling axis, we measured the phosphorylation of mTOR at Serine 2441 (S2441). The knockdown of CIP2A in H838 decreased the phosphorylation of mTOR while the overexpression caused the increased phosphorylation of mTOR (Fig. 5). These data suggested that CIP2A influenced the mTOR phosphorylation.

Since the two downstream substrates, eukaryotic initiation factor 4E-BP1 and P70 ribosomal S6 kinase 1 (P70S6K1), play important roles in cell activity, we also detected whether CIP2A is associated with any of mTOR downstream effectors. We found that depletion of CIP2A upregulated phosphorylation of both P70S6K1 and 4E-BP1.

## Discussion

Lung cancer causes the most cancer-related deaths in both men and women in the USA (3). Early diagnosis of lung cancer is technically difficult, since there are few noticeable symptoms in the patients at the onset of the disease (3). Although early diagnosis cannot guarantee recovery, appropriate treatment can be administered to prevent the advancement of this disease (8). Therefore, understanding the mechanisms underlying lung cancer tumorigenesis is key for the development of therapeutic targets.

Cancerous inhibitor of PP2A (CIP2A) was originally identified as a tumor-associated autoantigen in gastric and liver cancer (9). Previous studies have indicated that CIP2A is an oncoprotein to promote cancer cell proliferation through inhibition of c-myc associated PP2A phosphatase activity (10,13). The expression of CIP2A is tightly restricted to embryonic stage but often re-expressed in lung cancer tissues. Studies in our laboratory have also demonstrated that autoantibodies against this protein appear in high frequency in sera from lung cancer patients. The expression of this protein also has high frequency in lung cancer tissues. We proposed that CIP2A may be a potential biomarker to be incorporated into existing biomarker arrays for lung cancer diagnosis.

The functions of CIP2A in lung cancer have not been fully understood yet and its clinical relevance has not yet been established. According to our studies, we found the possible interaction between CIP2A and AKT signaling pathway, which may help to reveal the mechanism for CIP2A-promoted cancer progression. In the present study, we firstly attempted to address the relationship between CIP2A and AKT phosphorylation. The positive correlation between CIP2A and AKT phosphorylation in our results clearly demonstrated the possible regulation of AKT by CIP2A through growth-factor stimulation. This is not unexpected since previous studies had shown the role of CIP2A in promoting cell survival through downregulating AKT-associated PP2A phosphatase activity. Our studies not only confirmed the regulation under different treatments but also indicated an alternative way by which CIP2A promotes cancer progression, which is demonstrated by the altered cell proliferation by the introduction of mutant AKT (AKT CA and AKT DN). However, whether CIP2A physically interacts with AKT has not yet been explored.

Resistance to rapamycin of cancer cells is the major concern for the clinical use of this drug to treat cancer (21). Our preliminary data showed that CIP2A regulates the phosphorylation status of mTOR, the target of rapamycin and finally affects the sensitivity of cancer cells to the treatment; however, how this process is actually executed has yet to be determined. mTOR is the major component of the two complexes, mammalian target of rapamycin complex 1 and mammalian target of rapamycin complex 2 (mTORC1 and mTORC2). mTORC1 is a direct target of AKT while mTORC2 is the upstream kinase of AKT (22). mTORC1 is composed of mTOR, MLST8 and PRAS40, two of which, mTOR and PRAS40, are the targets of AKT. Therefore, CIP2A-mediated rapamycin sensitivity may be dependent on the regulation of mTOR, PRAS40 or both, which need further studies to be confirmed. In addition, our data also suggest that CIP2A would affect the phosphorylation of downstream targets of mTOR.

Collectively, our studies not only establish the correlation between CIP2A and AKT phosphorylation but also reveal novel functions of CIP2A in promoting AKT signaling cascade. Although further studies are required to increase the resolution for the identification of CIP2A-targeted AKT substrates as well as the role of CIP2A in chemoresistance, based on our preliminary data, we consider CIP2A a promising target for future drug design since it is an effective regulator to restrain cancer cell growth. The temporal expression of CIP2A will enable researchers to design specific drugs to target CIP2A, which may result in fewer side-effects. In addition, restoration of PP2A phosphatase activity is another therapeutic strategy in cancer treatment. Therefore, suppressing the expression of CIP2A may be an indirect way to reverse tumorigenic phenotypes and to treat cancer patients more efficiently.
